# Macrophages play a leading role in determining the direction of astrocytic migration in spinal cord injury via ADP-P2Y1R axis

**DOI:** 10.21203/rs.3.rs-2427082/v1

**Published:** 2023-01-09

**Authors:** Gentaro Ono, Kazu Kobayakawa, Hirokazu Saiwai, Tetsuya Tamaru, Hirotaka lura, Yohei Haruta, Kazuki Kitade, Kei-Ichiro Iida, Ken-Ichi Kawaguchi, Yoshihiro Matsumoto, Makoto Tsuda, Tomohiko Tamura, Keiko Ozato, Kazuhide Inoue, Dai-Jiro Konno, Takeshi Maeda, Seiji Okada, Yasuharu Nakashima

**Affiliations:** Department of Orthopaedic Surgery, Graduate School of Medical Sciences, Kyushu University, 3-1-1 Maidashi, Higashi-ku, Fukuoka, 812-8582, Japan; Department of Orthopaedic Surgery, Graduate School of Medical Sciences, Kyushu University, 3-1-1 Maidashi, Higashi-ku, Fukuoka, 812-8582, Japan; Department of Orthopaedic Surgery, Graduate School of Medical Sciences, Kyushu University, 3-1-1 Maidashi, Higashi-ku, Fukuoka, 812-8582, Japan; Department of Orthopaedic Surgery, Graduate School of Medical Sciences, Kyushu University, 3-1-1 Maidashi, Higashi-ku, Fukuoka, 812-8582, Japan; Department of Orthopaedic Surgery, Graduate School of Medical Sciences, Kyushu University, 3-1-1 Maidashi, Higashi-ku, Fukuoka, 812-8582, Japan; Department of Orthopaedic Surgery, Graduate School of Medical Sciences, Kyushu University, 3-1-1 Maidashi, Higashi-ku, Fukuoka, 812-8582, Japan; Department of Orthopaedic Surgery, Graduate School of Medical Sciences, Kyushu University, 3-1-1 Maidashi, Higashi-ku, Fukuoka, 812-8582, Japan; Department of Orthopaedic Surgery, Graduate School of Medical Sciences, Kyushu University, 3-1-1 Maidashi, Higashi-ku, Fukuoka, 812-8582, Japan; Department of Orthopaedic Surgery, Graduate School of Medical Sciences, Kyushu University, 3-1-1 Maidashi, Higashi-ku, Fukuoka, 812-8582, Japan; Department of Orthopaedic Surgery, Graduate School of Medical Sciences, Kyushu University, 3-1-1 Maidashi, Higashi-ku, Fukuoka, 812-8582, Japan; Department of Molecular and System Pharmacology, Graduate School of Pharmaceutical Sciences, Kyushu University, 3-1-1 Maidashi, Higashi-ku, Fukuoka, 812-8582, Japan; Kyushu University Institute for Advanced Study, Kyushu University, 744 Motooka Nishi-ku Fukuoka-shi Fukuoka 819-0395, Japan; Department of Immunology, Yokohama City University Graduate School of Medicine, 3-9 Fukuura, Kanazawa-ku, Yokohama,236-0004, Japan; Program in Genomics of Differentiation, NICHD, National Institutes of Health, Section on Molecular Genetics of Immunity, Building 6A, Room 2A01, 6 Center Drive, Bethesda, MD 20892, USA; Kyushu University Institute for Advanced Study, Kyushu University, 744 Motooka Nishi-ku Fukuoka-shi Fukuoka 819-0395, Japan; Greenpharma Research Center for System Drug Discovery, Kyushu University, 3-1-1 Maidashi, Higashi-ku, Fukuoka, 812-8582, Japan; Department of Energy and Materials, Faculty of Science and Engineering, Kindai University, Osaka 577-8502, Japan; Department of Orthopaedic Surgery, Spinal Injuries Center, 550-4 Igisu, Iizuka, Fukuoka, 820-8508, Japan; Department of Orthopaedic Surgery, Osaka University Graduate School of Medicine, 2-2 Yamada-oka, Suita, Osaka, Suita 565-0871, Japan; Department of Orthopaedic Surgery, Graduate School of Medical Sciences, Kyushu University, 3-1-1 Maidashi, Higashi-ku, Fukuoka, 812-8582, Japan

**Keywords:** Spinal cord injury, Macrophages, Astrocytes, Migration, P2Y1R

## Abstract

After spinal cord injury (SCI), inflammatory cells such as macrophages infiltrate the injured area, and astrocytes migrate, forming a glial scar around macrophages. The glial scar inhibits axonal regeneration, resulting in significant permanent disability. However, the mechanism by which glial scar-forming astrocytes migrate to the injury site has not been clarified. Here we show that migrating macrophages attract reactive astrocytes toward the center of the lesion after SCI. Chimeric mice with bone marrow lacking IRF8, which controls macrophage centripetal migration after SCI, showed widely scattered macrophages in injured spinal cord with the formation of a huge glial scar around the macrophages. To determine whether astrocytes or macrophages play a leading role in determining the directions of migration, we generated chimeric mice with reactive astrocyte-specific *Socs3*^−/−^ mice, which showed enhanced astrocyte migration, and bone marrow from *IRF8*^−/−^ mice. In this mouse model, macrophages were widely scattered, and a huge glial scar was formed around the macrophages as in wild-type mice that were transplanted with *IRF8*^−/^ bone marrow. In addition, we revealed that macrophage-secreted ATP-derived ADP attracts astrocytes via the P2Y1 receptor. Our findings revealed a mechanism in which migrating macrophages attracted astrocytes and affected the pathophysiology and outcome after SCI.

## Introduction

Although spinal cord injury (SCI) causes severe disability, effective treatments have not been established^1^. When the blood-spinal cord barrier is disrupted by mechanical injury, inflammatory cells, such as neutrophils and macrophages infiltrate into the spinal cord^2^, and these cells secrete inflammatory cytokines, such as interleukin (IL)-1α, IL-1β, IL-6, and tumor necrosis factor (TNF)-α, causing secondary damage^2^. After that, extracellular matrix deposition and astrocyte migration occur, and scar tissue is formed. The extracellular matrix and inflammatory cells are located at the center of the injury, and astrocytes migrate toward them to form a glial scar that serves as a physical barrier. Since this barrier prevents axonal regeneration, elucidation of the mechanism of glial scar formation is critical for developing new treatments for SCI.

We have previously reported that Stat3 regulates astrocyte migration^3^. However, the factors that attract astrocytes to the injury site remain unknown, although astrocyte migration is one of the most important processes in glial scar formation. In this study, we attempted to identify factors that attract astrocytes to the injury center by focusing on macrophages inside the glial scar. The proportion of macrophages at the injury center varies over time. Especially after the subacute stage, when the glial scar is formed, the infiltration of macrophages is particularly pronounced (7 days post-injury)^4^, suggesting that macrophages may also be involved in the formation of the glial scar. We hypothesized that macrophages might also be involved in the appearance of glial scars. We reported that in *IRF8*^−/−^ mice, in which macrophages are widely scattered after SCI due to impaired migration, larger glial scars were formed in comparison to wild-type mice^5^. In the central nervous system (CNS), astrocytes form close intercellular communication with other cell types, such as neurons and microglia, in which purine receptors play a significant role, for example, in regulating homeostasis, with consequences for synaptic transmission, and higher-order cognitive processes^6,7^. In addition, macrophages have been shown to attract other macrophages by secreting ATP, the ligand for the purine receptor^8^. Therefore, we hypothesized that macrophages also induce astrocytes via purine receptors. In this study, we demonstrated the effect of macrophages on astrocyte migration after SCI, which is critical for glial scar formation.

## Results

### Macrophages attract astrocytes in SCI

**Figure 1:**
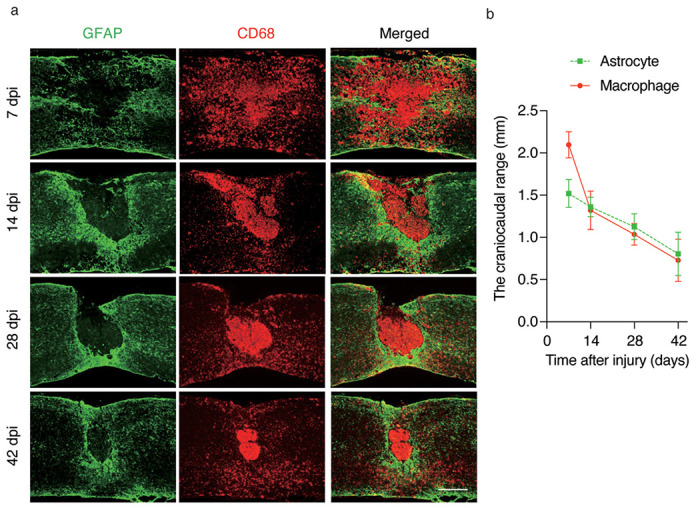
Temporal changes of macrophages and astrocytes in spinal cord injury. (a) Macrophage migration occurred after SCI, followed by glial scar formation by astrocytes at 7-14 days post-injury (dpi). Scale bars: 500 μm. (b) The quantitative analysis of the craniocaudal range of astrocytes in the glial scar and macrophages (n=6 per group).

**Figure 2:**
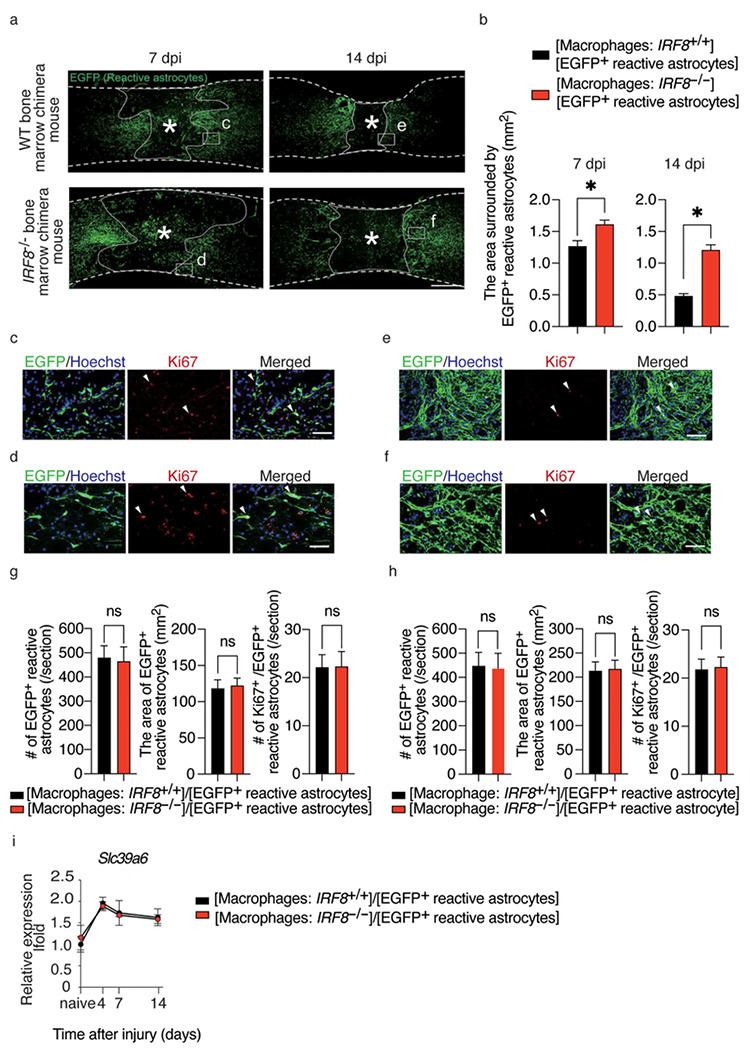
Inhibition of macrophage migration results in larger glial scars. (a) Change in the distribution of EGFP^+^ reactive astrocytes over time in the injured spinal cord of [Reactive astrocytes: *Nes-Cre-EGFP*^+^/ Macrophages: WT] and [Reactive astrocytes: *Nes-Cre-EGFP*^+^/ Macrophages: *IRF8*^−/−^] mice. [Reactive astrocytes: *Nes-Cre-EGFP*^+^/ Macrophages: *IRF8*^−/−^] mice had larger glial scars. Scale bar: 500 μm. (b) The quantitative analysis of the area surrounded by EGFP-positive cells: reactive astrocytes. There were significant differences between [Reactive astrocytes: *Nes-Cre-Socs3*^−/−^*EGFP*^+^/ Macrophages: WT] and [Reactive astrocytes: *Nes-Cre-Socs3*^−/−^*EGFP*^+^/ Macrophages: IRF8^−/−^] mice at 7 days and 14 dpi (n=6 per group at each time point). (c) - (f) High magnification field images of Figure.2 a are shown. Scale bars: 100 μm. (g) The quantitative analysis of the number of reactive astrocytes, the area of astrocyte cell bodies, and a proliferation assessment by Ki67 staining at 7 dpi. No significant differences were found in any of the parameters (n=6 per group). (h) The quantitative analysis of the number of reactive astrocytes, the area of astrocyte cell bodies, and a proliferation assessment by Ki67 staining at 14 dpi. No significant differences were found in any of the parameters (n=6 per group). (i) The time course of the *Slc39a6* expression in the injured spinal cord determined by real-time RT-PCR in [Reactive astrocytes: *Nes-Cre-Socs3*^−/−^EGFP^+^/ Macrophages: WT] and [Reactive astrocytes: *Nes-Cre-Socs3*^−/−^*EGFP*^+^/ Macrophages: *IRF8*^−/−^] mice. (n=6 per group at each time point). Each group was normalized to *Gapdh* values. There were no significant differences between [Reactive astrocytes: *Nes-Cre-Soc3*^−/−^*EGFP*^+^/ Macrophages: WT] and [Reactive astrocytes: *Nes-Cre-Socs3*^−/−^*EGFP*^+^/ Macrophages: *IRF8*^−/−^] mice. *p* < 0.05, Wilcoxon rank-sum test. Error bars indicate the SEM.

After SCI, macrophages infiltrated into the injury site and migrated toward the epicenter with the migration of reactive astrocytes toward the peri-injury area^5,9^. Initially, scattered macrophages and astrocytes migrate toward the epicenter over time as well, but eventually, they are located in different sites, with macrophages in the center of the lesion and astrocytes at the margin of the injury (Fig. 1a, b). These results suggest that macrophage migration may affect astrocyte migration. To investigate what happens to astrocytes when macrophage migration is impaired, a bone marrow chimera mouse model was created by transplanting bone marrow from *IRF8*^−/−^ mice (which have been reported to show impaired macrophage migration)^5^ into *Nes-Cre-EGFP* mice, in which reactive astrocytes expressed EGFP. Using the chimeric mice models, we compared the scar area at 7 and 14 days after SCI and found that the glial scar area in mice with impaired macrophage migration was significantly greater than that in the mice with normal macrophage migration at both time points (Fig. 2a, b). These results suggest that the migration of macrophages influences the migration of reactive astrocytes and subsequent astrocytic scar formation. In addition, we investigated whether the intrinsic nature of astrocytes was altered with impairment of macrophage migration. First, since astrocytes are known to polymerize at the injury margins in scar formation, we evaluated the effect on polymerization via cell density. We found no significant difference in cell density at either 7 or 14 days post-injury, indicating that loss of *IRF8* in macrophages had no significant effect on astrocyte polymerization (Fig. 2c–h). Next, since activated astrocytes are hypertrophied, we investigated whether astrocyte activity was affected by assessing the area of astrocyte cell bodies. As a result, the area of EGFP^+^ astrocytes was not altered by impaired macrophage migration, suggesting that impaired macrophage migration had no significant effect on the cellular activation of reactive astrocytes (Fig. 2c–h). Finally, the proliferative capacity of astrocytes was evaluated by Ki67 staining. No significant differences were found in proliferation between astrocytes in these two chimeric mice (Fig. 2c–h). These findings suggest that the macrophages attract astrocytes extrinsically, without altering their proliferative potential. We previously reported that *Slc39a6* is involved in the Stat3-mediated migration mechanism of reactive astrocytes^3^. Therefore, we assessed the expression of *Slc39a6* and found no significant difference (Fig. 2i). This result suggests that the effect of macrophages on astrocyte migration is not mediated by Stat3 in astrocytes. These findings indicate that IRF8-mediated macrophage migration affects reactive astrocyte migration without changing the proliferation, activation, or Stat3-mediated migration capacity of reactive astrocytes.

#### Macrophage migration is the leading factor in SCI.

**Figure 3:**
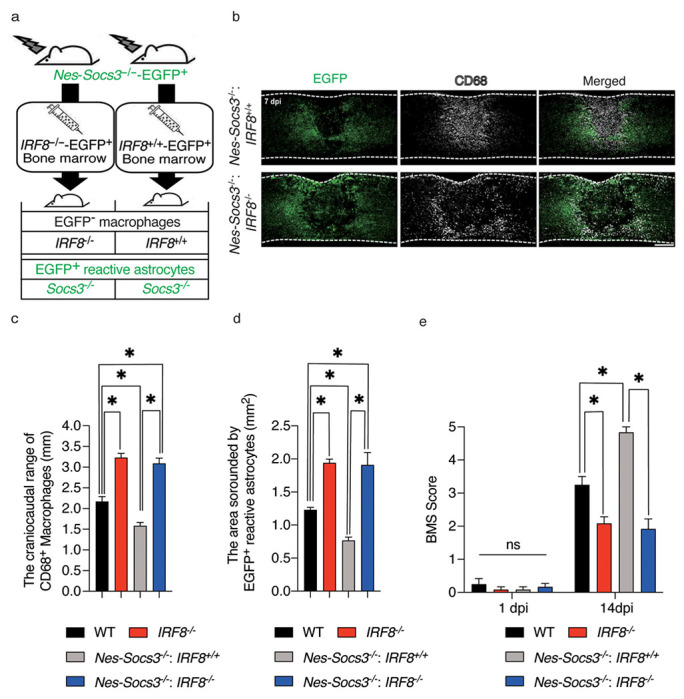
Impaired macrophage migration disturbs migration of genetically promoted migration of astrocytes after SCI. (a) A schematic illustration of the creation of bone marrow chimeric mice. (b) Immunostaining of spinal cord injury in [Reactive astrocytes: *Nes-Cre-Soc3*^−/−^*EGFP*^+^/ Macrophages: WT] and [Reactive astrocytes: *Nes-Cre-Soc3*^−/−^*EGFP*^+^/ Macrophages: *IRF8*^−/−^] mice. Scale bar: 500μm. (c) The quantitative analysis of the extent of macrophage migration. The lack of Socs3 in reactive astrocytes narrows the range of macrophage migration, while the lack of IRF8 widens the range of macrophage migration (n=6 per group). (d) The quantitative analysis of the area surrounded by EGFP-positive cells: reactive astrocytes. There were significant differences in the area between [Reactive astrocytes: *Nes-Cre-Soc3*^−/−^*EGFP*^+^ Macrophages: WT] and [Reactive astrocytes: *Nes-Cre-Soc3*^−/−^*EGFP*^+^/ Macrophages: *IRF8*^−/−^ mice at 7 days post-injury (n=6 per group). (e) The time course of motor function score after SCI. Significant differences were only seen at 14 dpi (n=6 per group). *p* < 0.05, one-way ANOVA with the Tukey-Kramer post hoc test. Error bars indicate the SEM.

In a previous study, we reported that the rapid migration of reactive astrocytes leads to the early accumulation of macrophages using *Nes-Socs3*^−/−^-*EGFP*^*+*^ mice, in which *Socs3* gene in reactive astrocytes was deleted^3^. In contrast, the poor migration of *IRF8*^−/−^ macrophages was accompanied by the impaired migration of reactive astrocytes (Fig. 2a, b). These facts suggest the possibility of interaction between infiltrating blood-derivedmacrophages and reactive astrocytes after SCI. To further investigate whether the migration of *IRF8*^−/−^ macrophages could affect the migration of reactive astrocytes, we generated two additional chimeric mice: [macrophages; *IRF8*^*+/+*^]/[reactive astrocytes; *Nes-Socs3*^−/−^-*EGFP*^*+*^ and [macrophages; *IRF8*^−/−^]/[reactive astrocytes; *Nes-Socs3*^−/−^-*EGFP*^*+*^] using EGFP-negative *IRF8*^*+/+*^ or *IRF8*^−/−^ bone marrow cells with *Nes-Socs3*^−/−^-*EGFP*^*+*^ recipient mice (Fig. 3a). In the former chimeric mice, *IRF8*^*+/+*^ macrophages showed extremely fast migration with the rapid migration of *Nes-Socs3*^−/−^ reactive astrocytes after SCI, as described in our previous study (Fig. 3b–d)^3^. However, even with *Nes-Socs3*^−/−^-*EGFR*^*+*^ reactive astrocytes, the migration disorder of *IRF8*^−/−^ macrophages was not rescued in the latter chimeric mice (Fig. 3b–d). Instead, the *Nes-Socs3*^*−/−*^-*EGFP*^*+*^ reactive astrocytes with *IRF8*^−/−^ macrophages migrated more slowly than those with *IRF8*^*+/+*^ macrophages (Fig. 3b, d). These results mean that while the migration of macrophages and reactive astrocytes interact with one another, the migration of macrophages is more strongly affected by macrophage IRF8 than by the migration of reactive astrocytes. We further examined the interaction between the migration of macrophages and reactive astrocytes on functional recovery after SCI. There was no significant deference among WT, *IRF8*^−/−^ and these chimeric mice before SCI, or at 1 day post-injury (Fig. 3e). However, at 14 days post-injury, although the former chimeric mice with WT macrophages and *Nes-Socs3*^−/−^ reactive astrocytes showed significantly ameliorated functional recovery in comparison to WT mice, the latter chimeric mice with *IRF8*^−/−^ macrophages and *Nes-Socs3*^−/−^ reactive astrocytes exhibited greater deterioration of the motor functional recovery in comparison to WT and the former chimeric mice (Fig. 3e). These findings revealed that, for motor recovery after SCI, autonomous migration of macrophages via IRF8 is more important than reactive astrocyte migration. Regarding astrocyte migration and macrophage migration, these findings clarified that macrophage migration plays a leading role in the interaction of their migration. In addition, Socs3 is a negative feedback factor for Stat3^10,11^, and it is known that Stat3 regulates reactive astrocyte migration via the Rho A small G protein^12^. In this study, the loss of IRF8 in macrophages also altered the migration of astrocytes with or without the expression of *Socs3.* This result also supports the hypothesis that the pathway by which macrophages attract astrocytes is not mediated by Stat3 (Fig. 2i).

### Macrophage-astrocyte Interactions Via P2y1 Receptors

**Figure 4:**
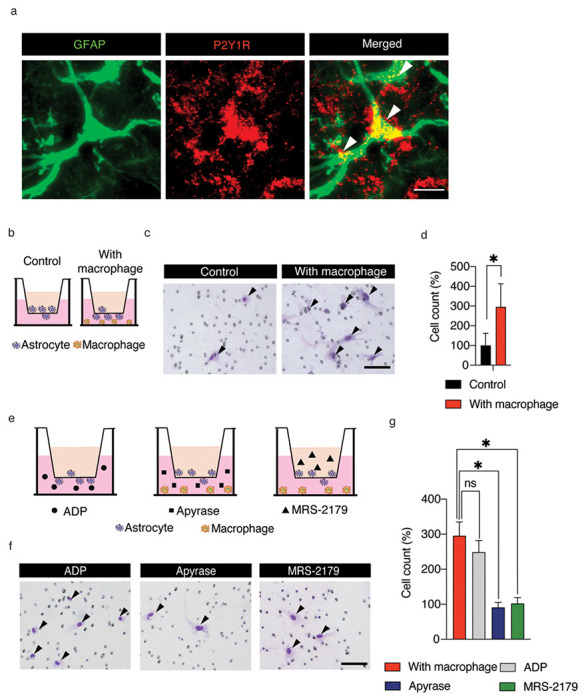
Macrophages attract astrocytes via the p2Y1R. (a) The expression of P2Y1R in the naive spinal cord. Scale bar: 500 μm. (b) A schematic illustration of the astrocyte transwell assay with/without macrophages. (c) The transwell assay of astrocytes in the control and macrophage groups. Diff-Quik staining images are representative of 2 independent experiments. Scale bar: 100 μm. (d) The comparison of the number of migrating cells in the control and macrophage groups (9 sections/3 wells per group). (e) A schematic illustration of the transwell assay to reveal the pathways by which macrophages attract astrocytes. (f) The transwell assay for astrocytes of the control and macrophage groups. Diff-Quik staining images are representative of 3 independent experiments. Scale bar: 100 μm. (g) The comparison of the number of migrating cells between macrophages, without macrophages/with ADP, with macrophages/with Apyrase, and with macrophages/with MRS-2179 (9 sections/3 wells per group). *p* < 0.05, one-way ANOVA with the Tukey–Kramer post hoc test. Error bars indicate the SEM.

To elucidate how macrophages affect the migration of astrocytes, we focused on P2Y1 receptor (P2Y1R) as a representative receptor in the migration pathways, because P2Y1R was reported to be involved in astrocyte velocity in traumatic brain injury^13^ and to regulate cell migration via the ADP-P2Y1R-MAP/ERK pathway^14^. Therefore, we hypothesized that ATP-derived ADP secreted by macrophages might attract astrocytes via P2Y1R. First, we performed immunostaining to confirm the expression of P2Y1R in the astrocytes of the injured spinal cord. As a result, P2Y1R was expressed in spinal cord astrocytes after SCI (Fig. 4a). Second, we performed a transwell assay using primary cultured astrocytes. To investigate whether macrophages attract astrocytes, we co-cultured the cells with macrophages (Fig. 4b). Co-culture with macrophages significantly increased the number of migrating astrocytes (Fig. 4c, d). Next, we performed three experiments to clarify the pathway by which macrophages attract astrocytes (Fig. 4e). First, we evaluated whether ADP attracts astrocytes. The number of migrating astrocytes was significantly increased when a medium with ADP was used, indicating that ADP attracts astrocytes (Fig. 4f, g). Second, to confirm whether the attraction of astrocytes by macrophages was due to the secretion of ADP, we performed co-culturing of macrophages and astrocytes with apyrase, an enzyme that degrades ADP (Fig. 4e). The results showed that the number of migrating astrocytes did not increase in the presence of apyrase, indicating that astrocyte migration is regulated by macrophage-derived ADP (Fig. 4f, g). Finally, to determine whether P2Y1R is the receptor on astrocytes on which ADP acts, we cocultured astrocytes and macrophages with MRS-2179, an antagonist of P2Y1R. With MRS-2179, the number of migrating astrocytes did not increase, indicating that astrocytes are attracted to macrophages by the ADP-P2Y1R pathway (Fig. 4f, g). Since ATP secreted from macrophages is degraded to ADP, macrophages would attract astrocytes via the ADP-P2Y1R axis, resulting in astrocyte migration toward the macrophages in the epicenter and astroglial scar formation after SCI.

## Discussion

In this study, we showed that the macrophages are essential for astrocyte migration using bone marrow chimeric mice. Impairment of macrophage migration also impaired the migration of astrocytes toward the center of injury. As a result, astrocytes became more widely distributed and the scar area increased with the impaired migration of macrophages. *Socs3*^−/−^ astrocytes, whose migration would be promoted with the migration of WT macrophages, did not migrate well toward the center of injury with impaired macrophage migration. These results indicate that macrophages play a leading role over astrocytes in the cell migration of macrophages and astrocytes after spinal cord injury. In addition, the co-culture of macrophages and astrocytes also showed that macrophages attract astrocytes. Using antagonists, we further elucidated that ADP derived from ATP secreted extracellularly by macrophages attracted astrocytes via P2Y1R. These findings indicated that macrophages affect the direction of astrocyte migration and are important clues to clarifying the pathophysiology of spinal cord injury.

In CNS injury, various cells interact with each other to produce dynamic changes. For example, microglia, neurons, and astrocytes have been reported to interact via P2Y receptors^13,15^. However, the influence of macrophages on astrocytes is not known. In this study, the great influence of macrophages on astrocyte migration, which occurs during the acute to subacute phase, was clarified. Since macrophages comprise the majority of inflammatory cells in the spinal cord scar and astrocytes are the major component of the glial scar, clarifying the interaction between macrophages and astrocytes is essential for the elucidation of the pathophysiology of SCI.

In addition, given that the glial scar, once formed in the subacute phase, is maintained into the chronic phase, the macrophage-derived ADP-astrocytic P2Y1R axis may also be involved in the mechanisms of glial scar maintenance^16^. We previously reported that scar forming astrocytes maintain the glial scar by changing the surrounding naïve astrocytes to scar-forming astrocytes^16^. However, it is not known how astrocytes are recruited to the scar. With regard to scar maintenance in the chronic phase, astrocytes may have been recruited to scar tissue via the macrophage-derived ADP-astrocyte P2Y1R axis.

In this study, we showed a mechanism in which macrophages attract astrocytes to the center of injury. Considering that astrocytes are arranged in a row at the outer edge of the scar and are rarely present inside the glial scar, astrocyte migration is not regulated only by the concentration gradient of ADP; some factor inside the scar stops astrocyte migration. However, the mechanism that stops the migration of astrocytes at the glial scar has not been elucidated. For example, PARP1 may be a candidate for the negative regulation of astrocyte migration. Stat3 is known to be essential for astrocyte migration^3^, and PARP1 has been reported to interact directly inhibit Stat3 phosphorylation by causing poly-ADP-ribosylation of Stat3^17^. The expression of PARP1 was upregulated in the spinal cord after SCI in comparison to the naïve spinal cord, as was previously shown in our RNA-seq study^18^. This result is consistent with the hypothesis that PARP1 negatively regulates astrocyte migration in SCI.

In conclusion, we demonstrated that the impairment of macrophage migration led to widespread astrocyte migration. Macrophages secrete ATP that is degraded to ADP, and astrocytes migrate toward macrophages via the ADP-P2Y1R pathway. Our findings provide deeper insight into the interaction between astrocytes and macrophages, and suggest a potential therapeutic target for SCI.

## Methods

### Animals

All study protocols involving mice were approved by the Committee of Ethics on Animal Experimentation of our institution and were conducted in accordance with the National Institutes of Health guidelines for the care and use of animals. All mice were housed in a temperature and humidity-controlled environment on a 12-hour light-dark cycle, and had *ad libitum* access to food and water.

All animal studies were approved by the Committee of Ethics on Animal Experimentation of our institute and conducted in accordance with ARRIVE guidelines (https://arriveguidelines.org). Every effort was made to reduce the number of animals used and to minimize their suffering.

*IRF8*^−/−^, *Nes-EGFP*^*+*^, *Nes-EGFP*^*+*^-*Socs3*^−/−^ mice were generated as described previously^3,19^. Bone marrow transplantation was performed as previously described^20^. Eight-week-old female C57BL/6 J mice were used as WT mice. Macrophagic IRF8-deficient chimeric mice were generated by transferring the bone marrow cells (BMCs) of *IRF8*^−/−^ mice into *Nes-EGFP*^*+*^ and *Nes-EGFP*^*+*^-*Socs3*^*−/−*^ recipient mice after irradiation, as previously described^20^.

### Spinal Cord Injury

Mice were anesthetized with pentobarbital (75 mg/kg intraperitoneally) and subjected to a contusion injury (70 kilodynes) at the 10th thoracic level using an Infinite Horizons Impactor (Precision Systems Instrumentation, Lexington, KY)^21^. After the injury, the overlying muscles were sutured, and the skin was closed with wound clips. During the period of recovery from anesthesia, the animals were placed in a temperature-controlled chamber until thermoregulation was re-established. The motor function was evaluated using a locomotor open-field rating scale, the BMS^22^.

### Primary Astrocyte Cultures

Purified primary astrocyte cultures were prepared from C57BL/6J mice, as described previously^3,23^. In brief, after removal of the meninges, on postnatal day 2, mouse brain tissues were minced and incubated in a rocking water bath at 37°C for 30 min in DMEM (08456-36, Nacalai Tesque Kyoto, Japan) in the presence of 0.25% trypsin (Nacalai Tesque Kyoto, Japan) and 4 mg/ml DNase I (Sigma, Saint Louis, MO). The dissociated cells were triturated with 0.25% FBS and centrifuged at 300 × *g* for 3 min. Following dilution with an astrocyte-specific medium: DMEM containing 10% FBS (Life Technologies, Carlsbad, CA) and 1% penicillin-streptomycin (Nacalai Tesque Kyoto, Japan), the cells were plated on Poly-L-Lysin coated T75 flask. After 7–10 days in a humidified CO_2_ incubator at 37°C, the T75 flask was set up on an orbital shaker to remove microglia at 180 rpm for 30 min. We added 20 ml of fresh astrocyte culture medium and then shook the flask at 240 rpm for 6 h to remove oligodendrocyte precursor cells. Astrocytes were detached from T75 flasks using 0.25% trypsin and were used for experiments.

### Primary Macrophage Culture

Purified primary macrophage cultures were prepared from C57BL/6J mice, as described previously^24^. In brief, after removing the muscles and tendons, Bone marrow cells were extracted from the femur. Following dilution with a macrophage-differentiation-specific medium: RPMI 1640 (Nacalai Tesque Kyoto, Japan) containing 10% FBS (Life Technologies, Carlsbad, CA), 1% penicillin-streptomycin (Nacalai Tesque Kyoto, Japan), and 40 ng/ml M-CSF (RSD, Minneapolis, MN 55413), the cells were plated on Poly-L-Lysin coated T25 flask. After 6 days in a humidified CO_2_ incubator at 37°C, the macrophages were detached from the T25 flask using EDTA (Nacalai Tesque Kyoto, Japan).

### Quantitative Reverse Transcription Polymerase Chain Reaction (Rt-pcr)

Total RNA was isolated from the astrocytes obtained from spinal cord tissue using the RNeasy Mini kit (Qiagen, Venlo, the Netherlands). cDNA was synthesized from the total RNA using PrimeScript Reverse Transcriptase (Takara, Tokyo, Japan) according to the manufacturer’s instructions. RT-qPCR was performed using primers specific to the genes of interest (Table 1) and a SYBR Premix Dimmer-Eraser (RR091A; Takara Bio, Shiga, Japan). Data were normalized to the level of glyceraldehyde-3-phosphate dehydrogenase (GAPDH). Real-time PCR was conducted using a CFX Connect Real-Time PCR Detection System (Bio-Rad, Hercules, CA).

### Histopathological Examinations

After animals were anesthetized and transcardially fixed with 4% paraformaldehyde (PFA; Millipore, Burlington, MA), the spinal cord was removed, dehydrated, and embedded in an optimal cutting temperature compound (Sakura Finetek Japan, Tokyo, Japan). The sections were mounted on MAS-coated slide glasses (Matsunami Glass, Kishiwada, Japan). On the other hand, astrocytes cultured *in vitro* were washed three times with PBS and fixed for 15 min in 4% PFA at room temperature. After washing three times with PBS, these sections and cells were used for immunofluorescent staining. Then the sections were stained with the following antibodies in a blocking solution overnight at 4°C: GFAP (1:500; rabbit; Dako, Santa Clara, CA; Z0334), GFAP (1:500; rat; Life Technologies, Carlsbad, CA; 130300), CD68 (1:1000; rat; Bio Rad, Hercules, CA; 94547), P2Y1R (1:500; rabbit; Allomone Labs, Jerusalem, ISR; APR-021). The primary antibodies were visualized with secondary antibodies conjugated to Alexa 488, 568, 647 (1:1000; Jackson ImmunoResearch, West Grove, PA). The nucleus was visualized with Hoechst 33258 (1:1000; Invitrogen, Waltham, MA). All images were captured using a BZ-X700 digital microscope system (Keyence, Osaka, Japan).

### Transwell Assay

The transwell assay was performed as described previously^25^. Using transwell inserts (No 354480; Corning; NY; 14831), a transwell assay was performed in 5 groups: (1) the control group: primary astrocytes in the inserts, with RPMI1640 in the well; (2) the macrophage group: primary astrocytes in the inserts, with RPMI 1640 with primary macrophages in the well; (3) the ADP group: primary astrocytes in the inserts, with RPMI 1640 with ADP (100μM; Oriental Yeast, Tokyo, Japan) in the well; (4) the Apyrase group: primary astrocytes in the inserts, with RPMI 1640 with primary macrophages and apyrase (10 U/ml; Sigma, Saint Louis, MO) in the well; and (5) the MRS-2179 group: primary astrocytes treated with MRS-2179 (10μM; Abcam, Cambridge, UK) in the inserts, with RPMI 1640 with primary macrophages in the well. After incubation for 24 hours and staining with Diff-Quick (Sysmex, Kobe, Japan), the number of migrating cells was counted in 9 sections/3 wells per group. The percentage increase in migrating cells compared to the control group was evaluated.

### Statistical Analyses

All statistical analyses were performed using the Graph Pad Prism software program, version 9.1.2 (GraphPad Software Inc., San Diego, CA.).

## Figures and Tables

**Table 1:** Primers used for quantitative RT-PCR

Gene Symbol	Accession Number	5– *ForwardPrimer* – 3	5 – *ReversePrimer* – 3
*Slc39a6*	NM_031168.2	TGAAGGCAGCACCAATAGCA-	GGCCTGGATGGTGATCATG
*Gapdh*	NM_008084.2	GACTTCAACAGCAACTCCCACTCT	GGTTTCTTACTCCTTGGAGGCCAT

## Data Availability

The datasets generated and/or analyzed in the current study are available upon request from the corresponding author.

## Abbreviations

SCI: Spinal cord injury
*IRF8*: Interferon Regulatory Factor 8
IL-1α: Interleukin-1α
IL-Iβ: Interleukin-1β
IL-6: Interleukin-6
TNF-α: Tumor necrosis factor -α
ATP: Adenosine triphosphate
ADP: Adenosine diphosphate
WT: Wild type
*Nes*: Nestin
*Socs3*: Suppressor of cytokine signaling-3
EGFP: Enhanced Green Fluorescent Protein
DMEM: Dulbecco’s Modified Eagle Medium
RPMI1640: Roswell Park Memorial Institute medium 1640
EDTA: Ethylenediaminetetraacetic acid
GFAP: Glial fibrillary acidic protein
CD68: Cluster of Differentiation 68
*Slc39a6*: Solute Carrier Family 39 Member 6
MAPK: Mitogen-activated protein kinase
ERK: Extracellular signal-regulated kinase
*Stat3*: Signal transducer and activator of transcription 3

## References

[1] McDonald J. W. & Sadowsky C. Spinal-cord injury. Lancet 359, 417–425 (2002). 11844532 10.1016/S0140-6736(02)07603-1

[2] Anjum A. Molecular Sciences Spinal Cord Injury: Pathophysiology, Multimolecular Interactions, and Underlying Recovery Mechanisms. doi:10.3390/ijms21207533. PMC7589539 33066029

[3] Okada S. Conditional ablation of Stat3 or Socs3 discloses a dual role for reactive astrocytes after spinal cord injury. (2006) doi:10.1038/nm1425. 16783372

[4] Orr M. B. & Gensel J. C. Spinal Cord Injury Scarring and Inflammation: Therapies Targeting Glial and Inflammatory Responses. Neurotherapeutics 15, 541 (2018). 29717413 10.1007/s13311-018-0631-6 PMC6095779

[5] Kobayakawa K. Macrophage centripetal migration drives spontaneous healing process after spinal cord injury. Sci. Adv vol. 5 https://www.science.org (2019). 10.1126/sciadv.aav5086 PMC6520026 31106270

[6] Illes P., Xu G.-Y. & Tang Y. Purinergic Signaling in the Central Nervous System in Health and Disease. Neurosci Bull 36,. 10.1007/s12264-020-00602-7 PMC7674523 33146814

[7] Burnstock G. Introduction to Purinergic Signalling in the Brain. Adv Exp Med Biol 1202, 1–12 (2020). 32034706 10.1007/978-3-030-30651-9_1

[8] Kronlage M. Autocrine purinergic receptor signaling is essential for macrophage chemotaxis. Sci Signal 3, (2010). 10.1126/scisignal.2000588 20664064

[9] Okada S. Conditional ablation of Stat3 or Socs3 discloses a dual role for reactive astrocytes after spinal cord injury. Nat Med 12, 829–834 (2006). 16783372 10.1038/nm1425

[10] Gao Q. Disruption of neural signal transducer and activator of transcription 3 causes obesity, diabetes, infertility, and thermal dysregulation. Proc Natl Acad Sci U S A 101, 4661 (2004). 15070774 10.1073/pnas.0303992101 PMC384803

[11] Mori H. Socs3 deficiency in the brain elevates leptin sensitivity and confers resistance to diet-induced obesity. Nat Med 10, 739–743 (2004). 15208705 10.1038/nm1071

[12] Renault-Mihara F. & Okano H. STAT3-regulated RhoA drives reactive astrocyte dynamics. Cell Cycle 16,1995–1996 (2017). 28933592 10.1080/15384101.2017.1377032 PMC5731407

[13] Shinozaki Y., Shibata K., Ikenaka K., Tanaka K. F. & Correspondence S. K. Transformation of Astrocytes to a Neuroprotective Phenotype by Microglia via P2Y 1 Receptor Downregulation. Cell Rep 19, (2017). 10.1016/j.celrep.2017.04.047 28494865

[14] Shen J. & Dicorleto P. E. ADP Stimulates Human Endothelial Cell Migration via P2Y 1 Nucleotide Receptor-Mediated Mitogen-Activated Protein Kinase Pathways. (2008) doi:10.1161/CIRCRESAHA.107.165795. 18174464

[15] Kofuji P. & Araque A. G-PROTEIN-COUPLED RECEPTORS IN ASTROCYTE-NEURON COMMUNICATION. Neuroscience 456, 71 (2021). 32224231 10.1016/j.neuroscience.2020.03.025 PMC8817509

[16] Tamaru T. Glial scar survives until the chronic phase by recruiting scar-forming astrocytes after spinal cord injury. Exp Neurol 359, 114264 (2023). 36336030 10.1016/j.expneurol.2022.114264

[17] Ding L. PARP1 suppresses the transcription of PD-L1 by poly(ADP-ribosyl)ating STAT3. Cancer Immunol Res 7, 136–149 (2019). 30401677 10.1158/2326-6066.CIR-18-0071

[18] Hara M. Interaction of reactive astrocytes with type I collagen induces astrocytic scar formation through the integrin–N-cadherin pathway after spinal cord injury. Nature Medicine 2017 23:7 23, 818–828 (2017). 10.1038/nm.4354 28628111

[19] Holtschke T. Immunodeficiency and chronic myelogenous leukemia-like syndrome in mice with a targeted mutation of the ICSBP gene. Cell 87, 307–317 (1996). 8861914 10.1016/s0092-8674(00)81348-3

[20] Rolls A. Two Faces of Chondroitin Sulfate Proteoglycan in Spinal Cord Repair: A Role in Microglia/ Macrophage Activation. PLoS Medicine | www 5, (2008). 10.1371/journal.pmed.0050171 PMC2517615 18715114

[21] Kobayakawa K. Acute hyperglycemia impairs functional improvement after spinal cord injury in mice and humans. Sci Transl Med 6, (2014). 10.1126/scitranslmed.3009430 25273098

[22] Ma M., Basso D. M., Walters P., Stokes B. T. & Jakeman L. B. Behavioral and histological outcomes following graded spinal cord contusion injury in the C57Bl/6 mouse. Exp Neurol 169, 239–254 (2001). 11358439 10.1006/exnr.2001.7679

[23] Schildge S., Bohrer C., Beck K. & Schachtrup C. Isolation and Culture of Mouse Cortical Astrocytes. JoVE (Journal of Visualized Experiments) e50079 (2013) doi:10.3791/50079. PMC3582677 23380713

[24] Ying W., Cheruku P. S., Bazer F. W., Safe S. H. & Zhou B. Investigation of Macrophage Polarization Using Bone Marrow Derived Macrophages. J Vis Exp (2013) doi:10.3791/50323. PMC3728835 23851980

[25] Marshall J. Transwell(^®^) invasion assays. Methods Mol Biol 769, 97–110 (2011). 21748672 10.1007/978-1-61779-207-6_8

